# Metallic-Cap Filling

**Published:** 1882-01

**Authors:** Stewart J. Spence


### METALLIC-CAP FILLING.

The position of the dental profession of to-day, as regards filling teeth, may be said to form an interrogation point. It is standing in an attitude of expectation, scanning the horizon for the arrival of the ideal filling. Metals expand and contract and let in moisture, or else they discolor and look offensive; the mineral pastes are friable externally and irritative internally; gutta-percha is most excellent internally, but too soft for outward use; neither is the ideal filling, and perhaps in the present state of dentistry there is no better practice than to combine the three, while we await the advent of that filling which will neither contract nor discolor, neither wear under mastication nor dissolve in oral fluids, of the color of tooth-substance, and as compatible with the pulp.

In the meantime it is well for us, with Laertes, to "hear each man's counsel." In offering mine, I shall define that particular mode of using in combination the three materials already mentioned which appeals to my understanding as the most appropriate in a large number of cases.

As metals contract from all points towards a center, it is evident that a metallic plug, in contraction, is not only drawn away from the side-walls of a cavity, but also from its floor and mouth, *and that this latter is in proportion to the length of the plug.* The "metallic-cap" admits only the minimum of contraction in this direction.

In excavating a cavity for gold filling, it has been customary to cut away a large area of firm enamel when overhanging subjacent decay. While this may be good practice when it eradicates suspicious-looking fissures, yet in numberless cases it bears the character of accommodation of the royal metal and unquestionably increases the contraction of the plug at the orifice, thus admitting the moisture which we are told converts our filled tooth into an electric battery, and which undoubtedly does increase the conductivity of the dentine, and thus tends to endanger the pulp. The metallic-cap manner of filling does not demand so great a sacrifice of enamel, and thus admits less shrinkage across the orifice.

The gist of the operation being advocated is simply this: Fill the bulk of the cavity to the inner edge of the enamel with some non-conducting material, and cap this with metal held in by a slight overhanging of the enamel walls.

I proceed in about this manner: When excavating the chamber, I avoid cutting the orifice as wide as the body of the cavity, and, by holding a fissure-burr at an angle of about ten degrees with the axis of the cavity, level away the enamel inwards on two opposing sides (this is to retain the metallic cap); then level *outwards*, very slightly, the sharp and ragged edge of enamel to insure against its chipping off under the mallet, and to permit that firm compaction of gold, when used adhesive, which an upward-inclined surface favors; then burnish the excised enamel; dry with heated air, and disinfect with carbolic acid diluted with alcohol; varnish with sandarac varnish sold at the depots, diluting it first with about fifty per cent. of alcohol. Place gutta-percha over the floor of the cavity, if it be large or the pulp in danger of irritation. I use a solution of the gum from which the chloroform has been permitted to evaporate, until the result is a material but little softer than ordinary cheese. In this condition it packs easily, and does not cling to the instrument or mouth of the cavity; it also hardens sufficiently almost immediately. This substratum being laid, the next step is to employ a material which will adhere so tenaciously to the dentine as to prevent the intervention of moisture. The mineral pastes present themselves. Using the phosphates of aluminum and zinc, I compress it firmly into the cavity until the lower edge of the enamel is reached—not merely lining the chamber, but filling it (almost)—so that should the metallic part come away, at any future period, the sub-filling is in a much better condition to remain firm than if the cavity were merely lined. If the enamel is thin, and it is desired to give a concave contour to the cap when finished, it is well to sink a slight concavity in the cement, so as to give the desired strength to the metallic cap. In a few minutes a cap of any metal may be put on. If amalgam be employed, I use it cold, condensing with the mallet, and scrupulously avoid over-filling the cavity, scooping away the mercury which rises to the surface; then, with a piece of spunk held in the foil-carrier, pat and press the surface until it is smooth, level with the orifice, and contoured according to the adjoining contour of tooth; avoiding, as much as possible, any angle at the union of the tooth and filling—such angle not only rendering burnishing difficult, but offering hospitality to the enemy.

The advantages of this course may be expressed thus:

The minimum contraction of a metallic filling is reached.

The minimum conductivity of a filling, when employing metal at its surface, is approximated.

Moisture is excluded from the dentine.

Discoloration of the tooth is obviated.

Bruising of the dentine by the mallet is avoided.—STEWART J. SPENCE, *in Dental Cosmos.*

